# Bioactivities of Compounds from *Elephantopus scaber*, an Ethnomedicinal Plant from Southwest China

**DOI:** 10.1155/2014/569594

**Published:** 2014-05-19

**Authors:** Jianjun Wang, Ping Li, Baosai Li, Zhiyong Guo, Edward J. Kennelly, Chunlin Long

**Affiliations:** ^1^College of Life and Environmental Sciences, Minzu University of China, 27 Zhong-Guan-Cun South Avenue, Haidian District, Beijing 100081, China; ^2^School of Chinese Materia Medica, Beijing University of Chinese Medicine, 11 Third Ring Road, Chaoyang District, Beijing 100029, China; ^3^Department of Biological Science, Lehman College, and Graduate Center, City University of New York, 250 Bedford Park Boulevard West, Bronx, NY 10468, USA; ^4^Kunming Institute of Botany, Chinese Academy of Sciences, 132 Lanhei Road, Kunming 650201, China

## Abstract

*Elephantopus scaber* is an ethnomedicinal plant used by the Zhuang people in Southwest China to treat headaches, colds, diarrhea, hepatitis, and bronchitis. A new **δ**-truxinate derivative, ethyl, methyl 3,4,3′,4′-tetrahydroxy-**δ**-truxinate (**1**), was isolated from the ethyl acetate extract of the entire plant, along with 4 known compounds. The antioxidant activity of these 5 compounds was determined by ABTS radical scavenging assay. Compound **1** was also tested for its cytotoxicity effect against HepG2 by MTT assay (IC_50_ = 60 **μ**M), and its potential anti-inflammatory, antibiotic, and antitumor bioactivities were predicted using target fishing method software.

## 1. Introduction

*Elephantopus* is a genus comprised of about 30 species worldwide, mainly distributed in South America, with only 2 species* E. scaber* and* E. tomentosus* found in Southwest China [1]. From 2008 to 2012, our ethnobotanical investigation in the traditional medicinal market, held during the Dragon-Boat Festival in the fifth month of the Chinese lunar calendar with a history of over 700 years, found that* Elephantopus scaber* L. (Asteraceae) is a common medicinal plant used by the Zhuang people in Jingxi County of Southwest China. The local Zhuang people use* E. scaber* commonly as a traditional herbal medicine to treat many ailments including headaches, colds, diarrhea, hepatitis, and bronchitis.

To date, 30 compounds have been reported from* E. scaber*, including 4 sesquiterpene lactones, 9 triterpenes, and 5 flavones. Previous bioactivity studies on* E. scaber* demonstrated that the extracts or compounds from this species have antibiosis, antivirus, and cytotoxicity activities [2]. The sesquiterpene lactones in particular have been explored for their anti-inflammatory and hepatoprotective activities [3], which partially proved the traditional knowledge of* E. scaber*.

**Figure 1:**
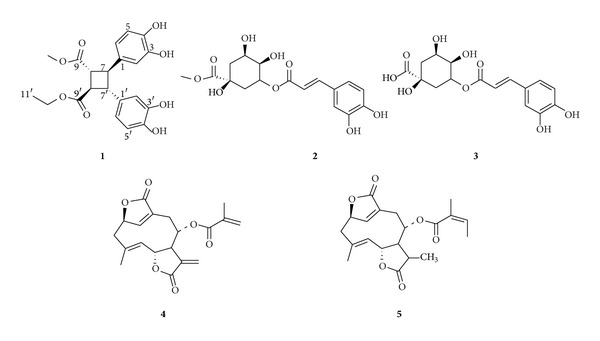
The chemical structures of compounds obtained from* Elephantopus scaber.*

In this paper, the isolation and structure elucidation of a new ethyl, methyl 3,4,3′,4′-tetrahydroxy-*δ*-truxinate (**1**, Figure 1) is reported, together with 4 known compounds, 5-*O*-caffeoylquinic acid (**2**) [4], chlorogenic acid methyl ester (**3**) [5], deoxyelephantopin (**4**), and isoscarbertopin (**5**) [6]. The radical scavenging activity of these 5 compounds was conducted using the ABTS method. The cytotoxicity effect against HpeG2 cell line of the new compound was determined by MTT assay, and the IC_50_ value (24.0 *μ*g/mL) was obtained. In addition, the potential activity of** 1**, calculated with target fishing, which used 3D structures of compounds to identify their interacting proteins by virtual screening [7], is also presented.

## 2. Materials and Method

### 2.1. Plant Material

The whole plant of* E. scaber* was collected from the traditional medicinal market during the Dragon-Boat Festival of Jingxi County (Guangxi), Southwest China, and identified by Professor Chunlin Long. A voucher specimen was deposited in the Herbarium of Minzu University of China, numbered 201006023.

### 2.2. Extraction and Isolation

The air-dried and ground whole plant of* E. scaber* (4.0 kg) was extracted with EtOH : H_2_O (90 : 10) at reflux for 3 × 3 h. The solvent was evaporated under reduced pressure to yield dark brown material (372.4 g). The latter was suspended in H_2_O (3 L) and individually partitioned with petroleum ether (3 × 3 L), Chloroform (2 × 3 L), EtOAc (3 × 3 L), and* n*-BuOH (3 × 3 L) to obtain petroleum ether (169.4 g), Chloroform (33.8 g), EtOAc (46.9 g), and* n*-BuOH (122.3 g) phase.

The EtOAc phase was separated by silica gel column chromatography (CC) eluted with CHCl_3_ : CH_3_OH in order of increasing polarity to give seven fractions on the basis of TLC. Fraction 3 was subjected to MCI CC eluted with CH_3_OH : H_2_O to seven fractions A_1_–A_7_. Fraction A_1_ was isolated by Sephadex LH-20, ODS CC (CH_3_OH : H_2_O = 44 : 56), and Si gel CC (CHCl_3_ : CH_3_OH = 14 : 1) successively to afford compound** 1** (24.0 mg). Fraction A_2_ was purified with Sephadex LH-20 to give six subfractions. Subfraction 2 was subjected to ODS CC (CH_3_OH : H_2_O = 30 : 70) and silica gel CC (CHCl_3_ : CH_3_OH = 12 : 1) successively to give compound** 3 **(17.0 mg). Subfraction 3 was subjected to ODS CC (CH_3_OH : H_2_O = 48 : 52) and Sephadex LH-20 to afford compound** 2** (27.0 mg).

The petroleum ether phase was separated by silica gel CC eluted with petroleum ether : EtOAc (100 : 1–0 : 100) to give ten fractions. Fraction 8 was purified by MCI CC using CH_3_OH : H_2_O (60 : 100–100 : 0) to afford four fractions B_1_–B_4_. Fraction B_2_ was subjected to Sephadex LH-20 and ODS CC (CH_3_OH : H_2_O = 83 : 17) to give compound** 4 **(9.0 mg). Fraction B_3_ was isolated by ODS CC (CH_3_OH : H_2_O = 80 : 20) and Sephadex LH-20 to give compound** 5 **(7.0 mg).

### 2.3. Antioxidant Assay

The antioxidant activity of compounds** 1**–**5** was evaluated with ABTS radical scavenging assay as described previously [8]. The IC_50_ was expressed as millimoles per liter (mM).

### 2.4. Cytotoxicity Assay

Compound** 1** was tested for cytotoxicity using a slightly modified MTT method [9]. Briefly 150 *μ*L (10 *μ*M, 20 *μ*M, 30 *μ*M, and 40 *μ*M) of samples was added to 96-well plate containing a confluent HepG2 cell monolayer in sextuplicate; 10 *μ*g/mL of norcantharidin (NCTD) and blank medium were used as the positive and control group, respectively. After a 72 h incubation at 37°C, 100 *μ*L MTT solution (5 mg/mL phosphate buffered saline) was added to each well, which was further incubated for 4 h for the formation of the formazan product. After removing the medium, 150 *μ*L DMSO was added to dissolve the formazan crystals. The optical density (OD) was measured at 550 nm with a microplate reader. The rate of inhibition was calculated by the following formula: rate of inhibition = (1 − sample OD)/control OD. The concentration causing inhibition of viable cells by 50% (IC_50_) was determined from a dose-response curve, which was based on triplicate measurements.

### 2.5. Virtual Screening

The potential activity of compound** 1 **was predicted by the “Target Fishing” functional model software (Discovery Studio). The target fishing process was conducted as follows. The DockScore energy function was utilized to minimize the energy of compound** 1 **conformation. Setting full minimization as minimization gave the smart conformation of compound** 1**. Then, pharmacophore search was set to be screened and profiled. Screen and profile was set to be ligand profiler. PharmaDB pharmacophores were set to be all. Conformation generation was set to be the best. Maximum conformation was set to be 200. Energy threshold was set to be 10. Saved conformations were set to be true, and other parameters were set to be default.

Top 14 candidate receptors were ranked according to the fit value (as shown in Table 2), which is based on force field approximation and specifically examined the compound internal energy and the compound-receptor interaction energy, which is taken as the sum of van der Waal force and electrostatic energy [10].

### 2.6. Ethyl, Methyl 3,4,3′,4′-tetrahydroxy-*δ*-truxinate

Light yellow oil; [*α*]_D_ −2.0°(*c* 0.018, MeOH); UV (in MeOH): *λ*_max⁡_ 284 and 228 nm; IR *ν*_max⁡_ ATR (cm^−1^): 3436, 2924, 2854, 1736, and 1600–1450; HRESIMS (*m/z*): 403.1286 [M+H]^+^; ^1^H NMR (300 MHz, CD_3_OD): *δ*_H_ 6.73 (4H, d-like), 6.62 (1H, t,* J* = 6.0, 3.0 Hz), 6.59 (1H, t,* J* = 6.0, 3.0 Hz), 4.19 (2H, q,* J* = 7.1 Hz), 3.73 (3H, s), 3.43 (1H, d-like,* J* = 2.9 Hz), 3.40 (1H, d-like,* J* = 2.5 Hz), 3.30 (1H, d-like,* J* = 3.1 Hz), 3.27 (1H, d-like,* J* = 3.5 Hz), and 1.26 (3H, t,* J* = 14.2, 7.1 Hz). ^13^C NMR (75 MHz, CD_3_OD): *δ*_C_ 173.5, 173.0, 145.0, 144.1, 132.8, 117.6, 115.0, 113.5, 60.7, 51.2, 50.2, 50.0, 46.2, 45.8, and 13.2.

## 3. Results

**Figure 2:**
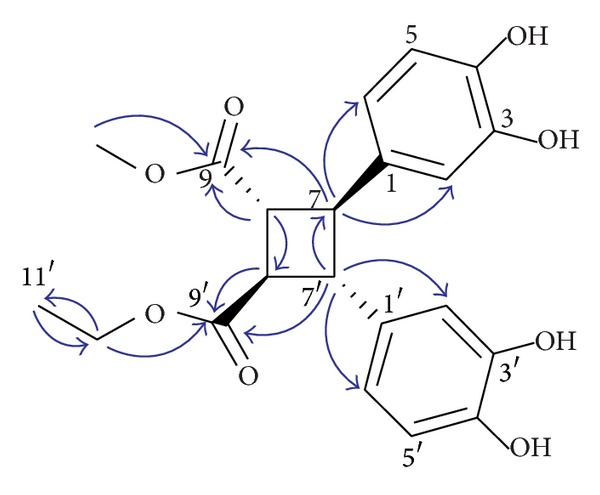
Selected HMBC (proton to carbon) correlations of compound** 1**.

Compound** 1 **(28.0 mg) was separated from the ethyl acetate extract of* E. scaber* whole plant as a light yellow oil. The molecular formula C_21_H_22_O_8_ was determined by the molecular ion observed at* m/z* 403.1359 [M+H]^+^ in the LC-TOF-MS (positive mode), which requires 11 degrees of unsaturation. The IR spectrum presented bands in the 1600–1450 cm^−1^, 1736 cm^−1^, 2854 cm^−1^, 2924 cm^−1^, and 3436 cm^−1^ region, which corresponded to aromatic, ester, methyl or methylene, and phenolic hydroxyl groups, respectively. The structure of compound** 1** was further elucidated by examination of its 1D ^13^C (75 MHz), DEPT (90° and 135°), and ^1^H (300 MHz) NMR spectra and HMQC, HMBC, COSY, and NOESY spectra in MeOH-*d*_4_. Only 13 carbon resonance signals and the other two carbon signals overlapped by the solvent carbon signals were found in ^13^C NMR and DEPT spectra, respectively, which suggested that there are many identical parts in this molecule. Further analysis of the ^13^C NMR spectrum of compound** 1 **suggested each signal of *δ*_C_ 145.0, 144.1, 132.8, 117.6, 115.0, and 113.5 is comprised of two overlapping carbon signals [*δ*_C_ 145.0 (C-3 and C-3′), 144.1 (C-4 and C-4′), 132.8 (C-1 and C-1′), 117.6 (C-6 and C-6′), 115.0 (C-5 and C-5′), and 113.5 (C-2 and C-2′)]; the other signals were assigned to two carbonyl carbon atoms [**δ** 173.5 (C-9) and 173.0 (C-9′)], one methoxy group (*δ*_C_ 60.7, C-10), one submethoxy (*δ*_C_ 51.2, C-10′), methyl (*δ*_C_ 13.2, C-11′), and four methine carbons [*δ*_C_ 50.2 (C-7′), 50.0 (C-7), 46.2 (C-8′), and 45.8 (C-8)]. From the number of unsaturations and carbons, these four methane carbons were deduced to be cyclobutane ring. From the analysis of ^1^H NMR spectrum of compound** 1**, two phenylpropanoid units were presented at *δ*_H_ 6.73 (4H, d-like, H-2, H-5, H-2′, H-5′), *δ*_H_ 6.62 (1H, t,* J* = 6.0, 3.0 Hz, H-6/H-6′), and 6.59 (1H, t,* J* = 6.0, 3.0 Hz, H-6′/H-6). Four methane carbons of the cyclobutane ring were observed at *δ*_H_ 3.43 (1H, d-like,* J* = 2.9 Hz, H-7), 3.40 (1H, d-like,* J *= 2.5 Hz, H-7′), 3.30 (1H, d-like,* J* = 3.1 Hz, H-8′), and 3.27 (1H, d-like,* J* = 3.5 Hz, H-8), and the relative configuration of the cyclobutane ring was determined by comparing the chemical shift of compound** 1** with reported ^1^H NMR data of other**δ**-truxinate derivatives [11]. Other signals of ^1^H NMR spectra were assigned to submethoxy [*δ*_H_ 4.19 (2H, q,* J* = 7.1 Hz, H-10′)], methoxy [*δ*_H_ 3.73 (3H, s, H-10)], and methyl [*δ*_H_ 1.26 (3H, t,* J* = 14.2, 7.1 Hz, H-11′)]. Meanwhile, the HMBC spectrum of compound** 1** presented the correlations from H-10 to C-9, H-8 to C-9 and C-8′, H-7 to C-2 and C-6, H-11′ to C-10′, from C-10′ to H-9′ and H-11′, from C-8′ to C-9′, and from C-7′ to H-2′ and H-6′, respectively (Figure 2). Consequently, the structure of compound** 1** was deduced to be ethyl, methyl 3,4,3′,4′-tetrahydroxy-**δ**-truxinate, which was further confirmed by HMQC, COSY, and NOESY spectra. This paper reports a new**δ**-truxinate derivative in* Elephantopus* genus for the first time. Compounds** 2**–**5** were identified, respectively, as 5-*O*-caffeoylquinic acid (**2**), chlorogenic acid methyl ester (**3**), deoxyelephantopin (**4**), and isoscarbertopin (**5**) by comparing their NMR and MS data with reported literature values.

The antioxidant activity of 5 compounds isolated from* E. scaber* was evaluated by the ABTS radical scavenging assay, and the results are presented as IC_50_ in Table 1. The most active radical scavengers were the new compound ethyl, methyl 3,4,3′,4′-tetrahydroxy-**δ**-truxinate (IC_50_ = 0.44 ± 0.039 mM). The other 2 quinic acid derivatives 5-*O*-caffeoylquinic acid and chlorogenic acid methyl ester also showed radical scavenging potential (IC_50_ = 0.96 ± 0.096 and 0.89 ± 0.140 mM, resp.), while the antioxidant activity of the other 2 sesquiterpene lactone compounds deoxyelephantopin and isoscabertopin was not detected. Comparing the structures of these 5 compounds, the different antioxidant activities were attributed to the existence of phenolic hydroxyl groups in compounds, which were supported by the previous reports [12].

**Figure 3:**
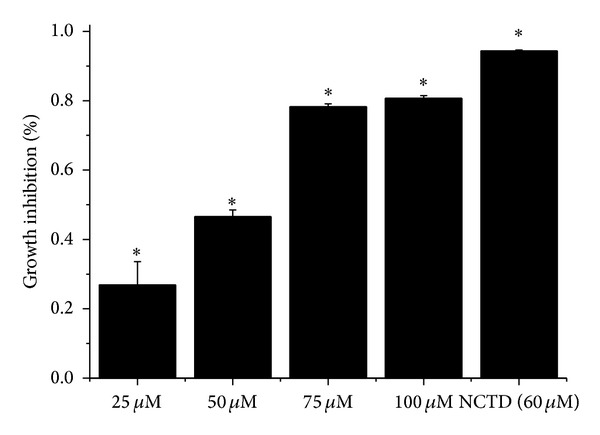
Growth inhibition of compound** 1** on HepG2 cell lines, where each value represents mean ± standard deviation of 6 replicates (*n* = 6), as compared to the positive control norcantharidin (60 *μ*M) (*P < 0.001 for comparison of control cell with cells treated with compound** 1** and NCTD); 72 h for the incubation period of cells after being treated with compound** 1** and NCTD.

Compound** 1 **was also tested for* in vitro* cytotoxicity against HepG2 cell line with norcantharidin (NCTD, 60 *μ*M) as positive control at 72 h incubation (Figure 3). Compound** 1 **exhibited a dose-response inhibition curve from 27% growth inhibition at 10 *μ*g/mL to 81% at 40 *μ*g/mL, demonstrating that it has significant and dose-dependent inhibition on the growth of HepG2 (IC_50_ = 60 *μ*M). Further work will be conducted on the mechanism by which compound** 1** induces apoptosis.

With the rapid development of computer-aided drug design (CADD), virtual screening technique has been used more and more widely in drug design and bioactivity screening of compounds [13]. The potential bioactivities of compound** 1 **have been predicted by the target fishing method which was based on the Discovery Studio software and Protein Date Bank (PDB) including over twelve thousand 3D macromolecular structure data determined experimentally by X-ray crystallography and NMR. The top 14 biological molecular targets ranked as the fit value (FV) are reported (Table 2).

Theoretically, FV > 3 means this target should be explored experimentally. The strongest activity of compound** 1** was predicted to be anti-inflammatory (FV = 4.05271) and anti-AIDS (FV = 3.25549), respectively (Table 2). Further experiments on the biological functions of** 1 **should be directed towards its potential anti-inflammatory, antibiotic, antivirus, and anticancer activities.

## 4. Discussion and Conclusion

With more and more present modern drugs discovered from traditional medical knowledge, the traditional knowledge is getting more extensive attention, which also led to the development of important drugs such as reserpine (a treatment for hypertension) podophyllotoxin (the base of an important anticancer drug), and vinblastine (used in the treatment of certain cancers) [46].

Previous studies showed that pulmonary oxidant stress can cause some disease conditions, such as acute lung injury, radiation injury, COPD (chronic obstructive pulmonary disease), and inflammation [47]. Meanwhile, previous clinical and experimental studies described that antioxidant supplementation including flavonoids and vitamins may inverse the oxidant-mediated cough depression by modulating the inflammatory process in lung disease [48, 49]. Interestingly, our work using ABTS assay demonstrated that compounds** 1**–**3** showed strong antioxidant activity, especially compound** 1 **(IC_50_ = 0.44 ± 0.039 mM). Moreover,* E. scaber* was also reported as the source of a number of sesquiterpene lactones, such as compounds** 4** and** 5**, which have shown significant contribution to the anti-inflammatory activity of plants [50]. Some of the sesquiterpenes from the genus* Elephantopus* have demonstrated significant anti-inflammatory as well as hepatoprotective activities and are being considered as drug lead compounds [3]. Based on the above analysis, we hypothesize that Zhuang people use this plant to treat headaches, bronchitis, and hepatitis,due to its anti-inflammatory and antioxidant effects.

According to the* in vitro* cytotoxicity assay with NCTD (60 *μ*M) as control group and activity virtual screening, compound** 1 **exhibited good (IC_50_ = 60 *μ*M) and dose-response inhibition on HepG2 cell line and potential anti-inflammatory, antibiotic, antivirus, and anticancer activities, which indicated that the further research of* E. scaber* could be focused on anticancer and anti-inflammatory activity. The present work further developed the usage of this traditional medicine plant.

## Supplementary Material


*Elephantopus scaber* is an ethnomedicinal plant used by the Zhuang people in Southwest China to treat headaches, colds, diarrhea, hepatitis, and bronchitis. A newσ-truxinate derivative, ethyl, methyl 3,4,3,4-tetrahydroxy-σ-truxinate (1), was isolated from the ethyl acetate extract of the entire plant, along with 4 known compounds. The isolation procedure and the NMR, mass, UV, IR spectrums are all shown herein as supplementary material

## Figures and Tables

**Table 1:** The radical scavenging activity of 5 compounds from* Elephantopus scaber*.

Compound	Name	IC_50_ (mM)^a^
**1**	Ethyl, methyl 3,4,3′,4′-tetrahydroxy-**δ**-truxinate	0.44 ± 0.039
**2**	5-*O*-caffeoylquinic acid	0.96 ± 0.096
**3**	Chlorogenic acid methyl ester	0.89 ± 0.140
**4**	Deoxyelephantopin	NR
**5**	Isoscarbertopin	NR
**6** ^b^	Trolox	1.33 ± 0.187

^a^The inhibition was recorded at 10 min of reaction (ABTS method) and IC_50_ value was measured using PROBIT model: PROBIT (*p*) = intercept + *BX* (covariates *X* are transformed using the base 10.000 logarithm). Each value corresponds to the mean and standard deviation of duplicates at five concentrations.

^b^Positive control group.

NR: No reaction at the conditions discribed.

**Table 2:** The potential bioactivity screening results of compound **1**.

Pharmacophore	Name of pharmacophore	Type	Fit value	Biological function(s)	Reference
2zb8-01-s	Prostaglandin reductase 2	Protein	4.05271	Inflammation	[14]
3kjs-01	Dihydrofolate reductase-thymidylate synthase	Protein	3.9615	Malarial parasites, anticancer, and inflammation	[15–17]
2uue-01	Cell division protein kinase 2	Protein	3.60758	Cell division	[18, 19]
2w4i-01-s	Glutamate racemase	Protein	3.55547	Antibiotics	[20–24]
3k6l-01	Peptide deformylase	Protein	3.51102	Antibiotic	[25, 26]
3md7-01	*beta*-lactamase-like	Protein	3.41887	Antibiotic	[27, 28]
2ovy-01	Phosphodiesterase 10A	Protein	3.41834	Schizophrenia and nervous system	[29–31]
3ac8-01	Protooncogene tyrosine-protein kinase LCK	Protein	3.39142	Antitumor	[32, 33]
3f7z-01	G17 glycogen synthase kinase-3-*beta *	Protein	3.30076	Antitumor and neurodegenerative disease	[34]
3cgy-01	Virulence sensor histidine kinase phoQ	Protein	3.28695	Antibiotic	[35, 36]
2qe5-01	RNA-directed RNA polymerase	Protein	3.28198	Antivirus	[37, 38]
1dvx-01	Transthyretin	Protein	3.27421	Antitumor and obesity	[39, 40]
2brc-01	ATP-dependent molecular chaperone HSP90	Protein	3.26648	Antitumor and antivirus	[41, 42]
1c1b-01-s	HIV-1 reverse transcriptase (A-chain)	Protein	3.25549	Anti-HIV	[43–45]
