# Supplementary material for: Bioactivities of Compounds from Elephantopus scaber, an Ethnomedicinal Plant from Southwest China
Source: Evid Based Complement Alternat Med. 2014 May 19;2014:569594. doi: 10.1155/2014/569594 (PMC4055671; doi:10.1155/2014/569594)

**Supplementary Materials**

**Bioactivities of compounds from *Elephantopus scarber*,**

**An ethnomedicinal plant from southwest China**

Jianjun Wang ^a^, Ping Li^a^, Baosai Li^c^, Zhiyong Guo^a^, Edward J. Kennelly ^a,b^, Chunlin Long ^a,d^,*

^a^ *College of Life and Environmental Sciences, Minzu University of China, 27 Zhong-Guan-Cun South Avenue, Haidian District, Beijing 100081, China*

^b^ *Department of Biological Science, Lehman College, and Graduate Center, City University of New York, 250 Bedford Park Boulevard West, Bronx, NY 10468, United States*

*^c^ School of Chinese MateriaMedica,* *Beijing University of Chinese Medicine, 11 Third-Ring-Road, Chaoyang District, Beijing 100029, China*

^d^ *Kunming Institute of Botany, Chinese Academy of Sciences, 132 Lanhei Road, Kunming 650201, China*

Corresponding author: Tel/fax: +86 10 68930381.

*E-mail address*: [long@mail.kib.ac.cn](mailto:long@mail.kib.ac.cn) (C.-L. Long)

1. **The isolation process of *Elephantopus scarber***

§

***E. scarber* - Whole plant** (4.0 kg)

Fr1-7

Fr9-10

Fr8

EtOH-H_2_O(90:10), Reflux (3×3 h)

Petroleum ether extract (169.4 g)

Chloroform extract (33.8 g)

Ethyl acetate extract (46.9 g)

n-butanol extract

(122.3 g)

Si gel CC- petroleum ether: EtOAc (100:1-0:100)

Si gel CC- petroleum ether: EtOAc (100:1-0:100)

B2

B3

B4

① ODS CC (CH_3_OH:H_2_O=80:20)

② Sephadex LH-20

Compound **4**

(9.0 mg)

Compound **5**

(7.0 mg)

Si gel CC- CHCl_3_:CH_3_OH (100:1-0:100)

Fr1-2

Fr3

Fr4-7

Si gel CC- petroleum ether: EtOAc (100:1-0:100)

A1

A2

A3-7

① Sephadex LH-20

② ODS CC (CH_3_OH:H_2_O=44:56)

③ Si gel CC (CHCl3:CH3OH=14:1)

Compound **1**

(24.0 mg)

Sub1

Sub2

Sub3

Sub4-6

① ODS CC (CH_3_OH:H_2_O=30:70)

② Si gel CC (CHCl_3_:CH_3_OH=12:1)

① ODS CC (CH_3_OH:H_2_O=48:52)

② Sephadex LH-20

Compound **3**

(17.0 mg)

Compound **2**

(27.0 mg)

B1

① Sephadex LH-20

② ODS CC (CH_3_OH:H_2_O=83:17)

1. **NMR specrum of compound 1:**

^1^H-Spectrum:


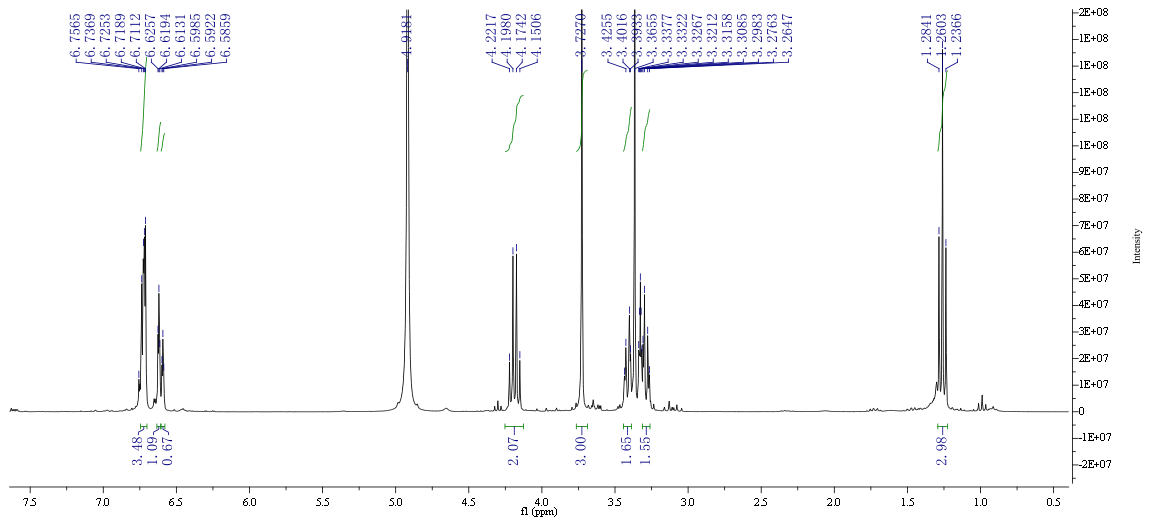


^13^C-Spectrum:


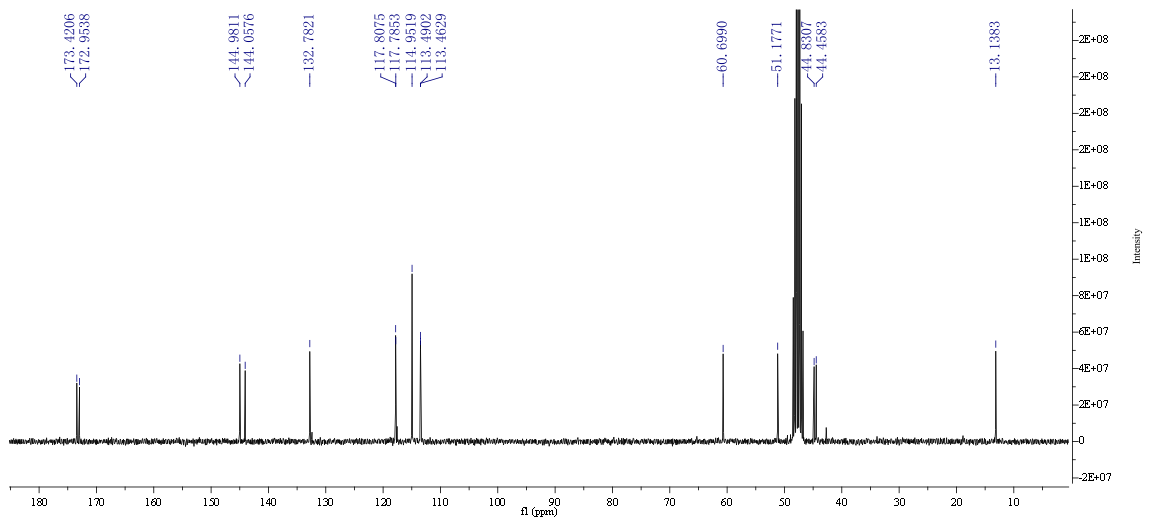


DEPT-135 Spectrum:


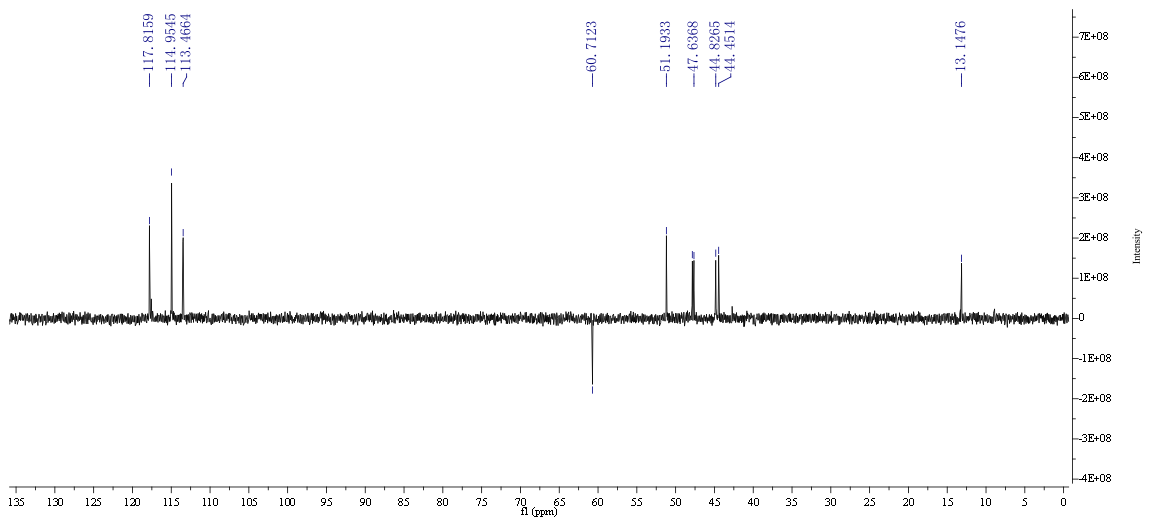


DEPT-90 Spectrum:


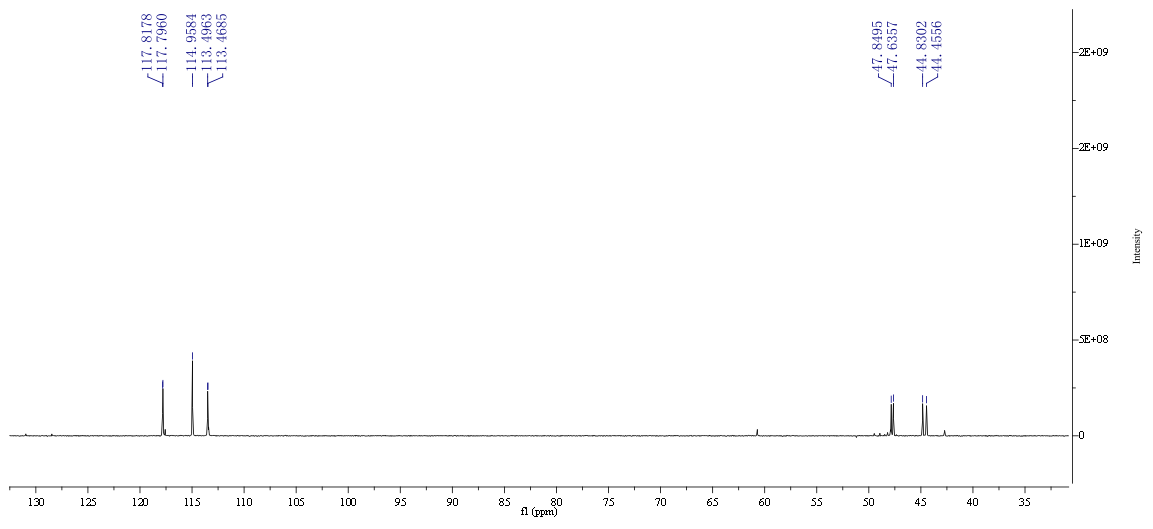


HSQC-Spectrum:


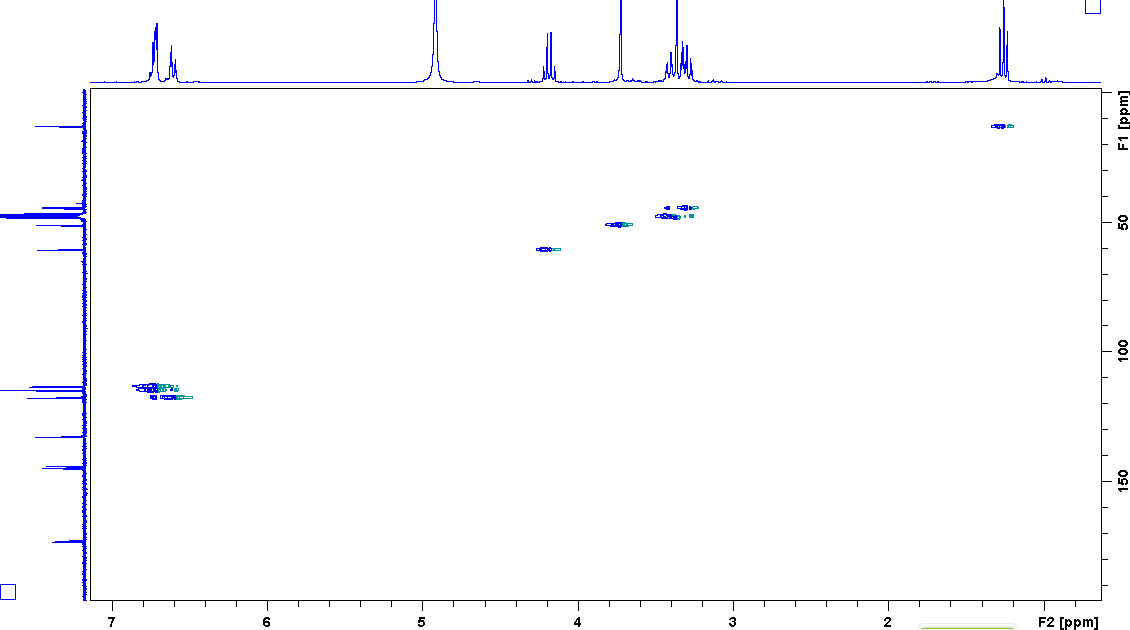


HMBC-Spectrum:


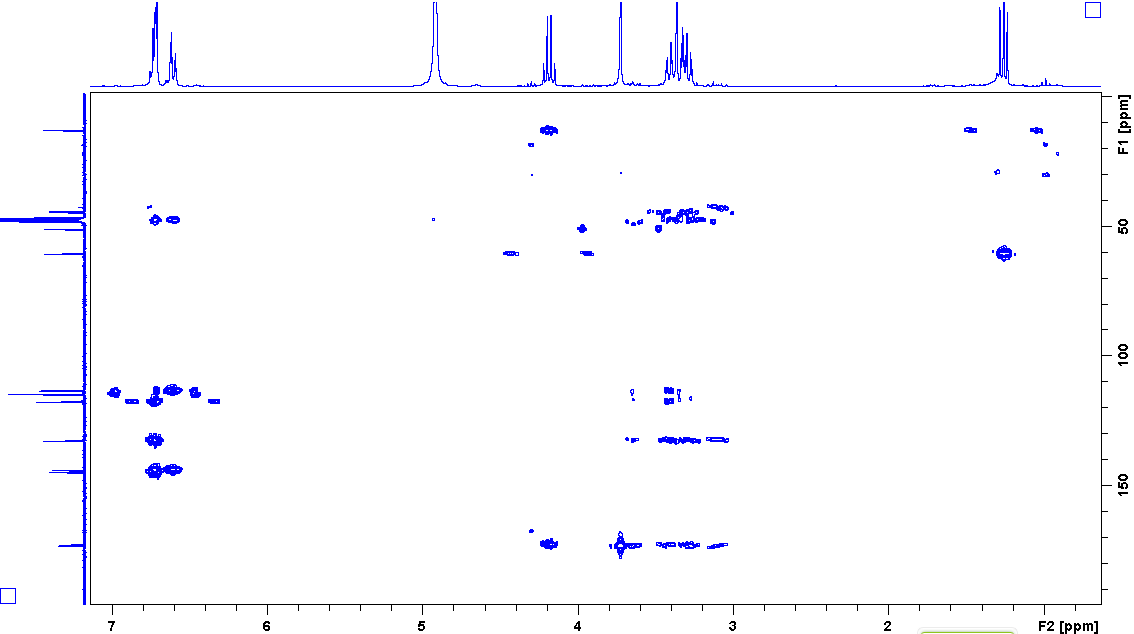


Cosy-Spectrum:


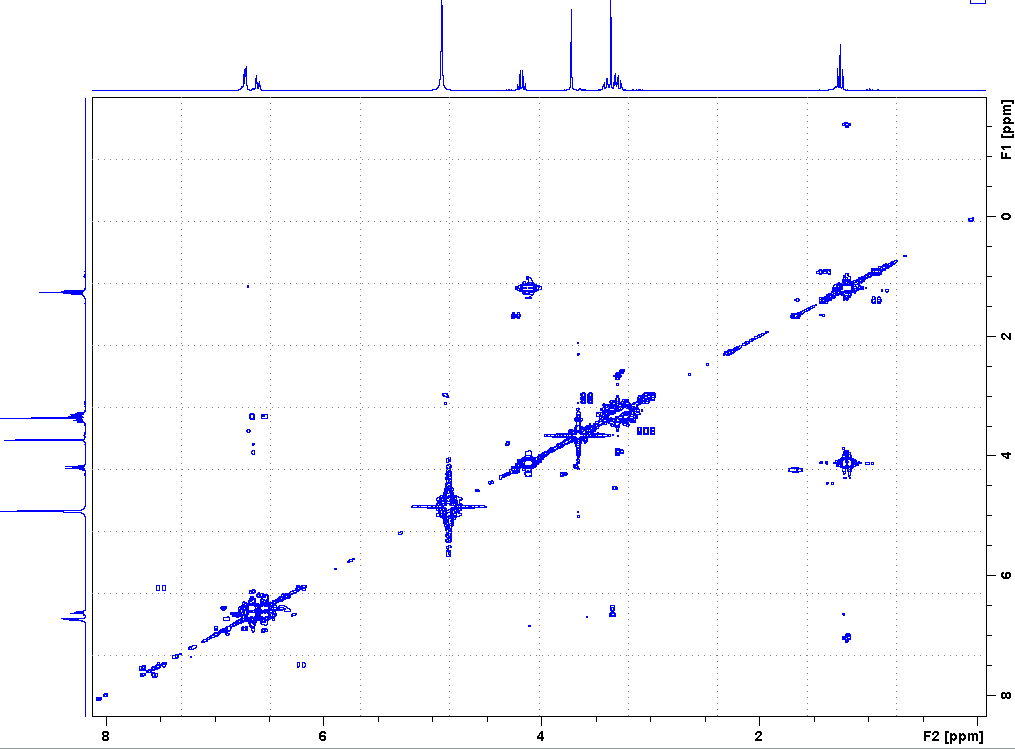


Noesy-Spectrum:


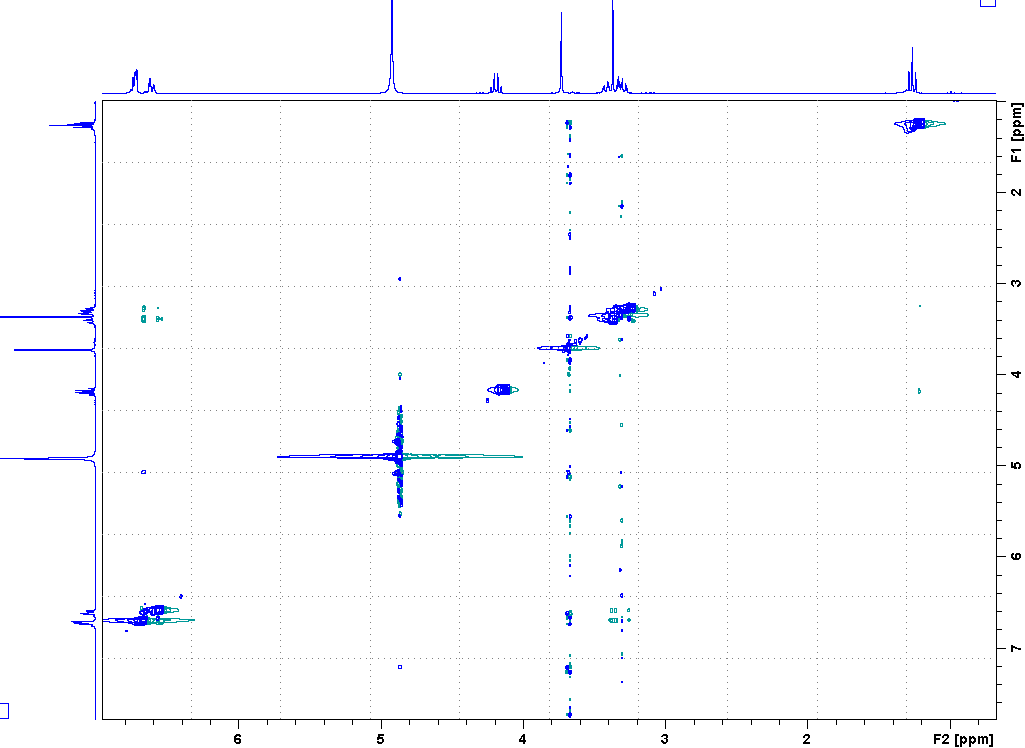


HPLC-TOF-MS:

UV:


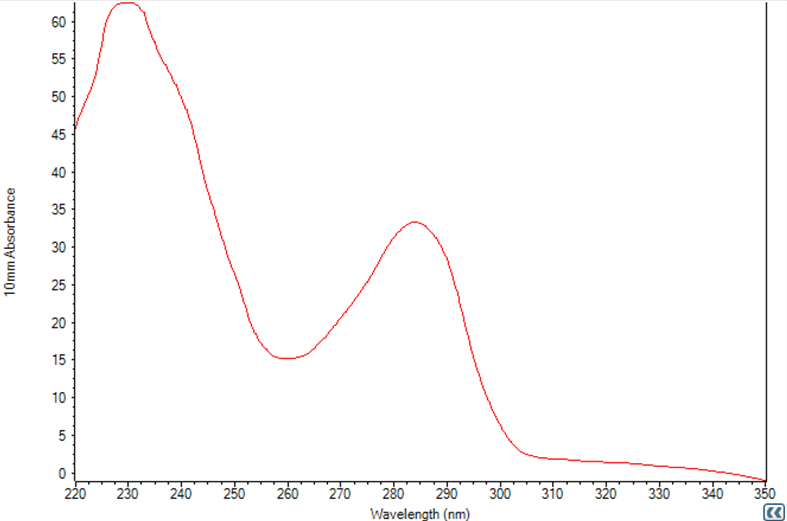


IR:


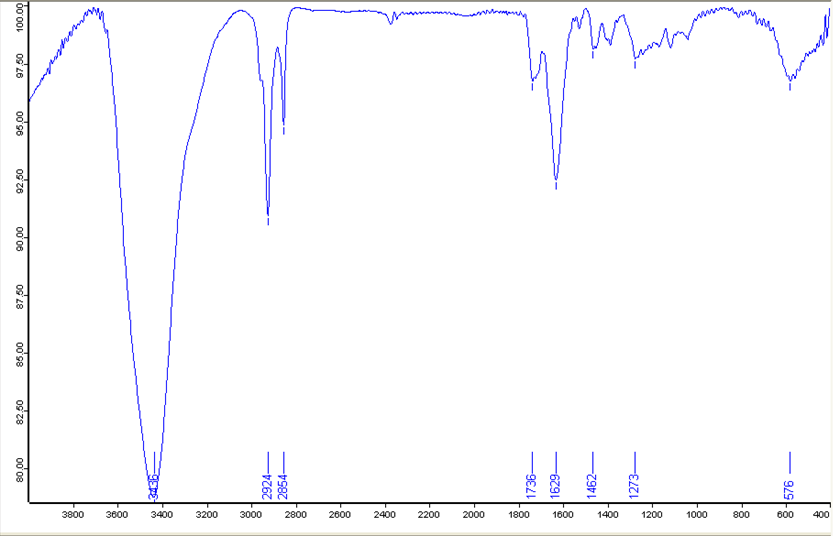

Supplement: Supplementary file 1 — Elephantopus scaber is an ethnomedicinal plant used by the Zhuang people in Southwest China to treat headaches, colds, diarrhea, hepatitis, and bronchitis. A newσ-truxinate derivative, ethyl, methyl 3,4,3,4-tetrahydroxy-σ-truxinate (1), was isolated from the ethyl acetate extract of the entire plant, along with 4 known compounds. The isolation procedure and the NMR, mass, UV, IR spectrums are all shown herein as supplementary material [file 569594.f1.doc]
